# Overlap syndrome of primary biliary cholangitis and primary sclerosing cholangitis: two case reports

**DOI:** 10.1186/s13256-023-03908-y

**Published:** 2023-04-28

**Authors:** Haythem Yacoub, Sarra Ben Azouz, Hajer Hassine, Habiba Debbabi, Dhouha Cherif, Feriel Ghayeb, Seif Boukriba, Héla Kchir, Nadia Maamouri

**Affiliations:** 1 Gastroenterology B Department, La Rabta Hospital, Tunis, Tunisia; 2 Radiology Department, La Rabta Hospital, Tunis, Tunisia

**Keywords:** Overlap syndrome, PBC, PSC, UDCA

## Abstract

**Background:**

Overlap syndrome between primary biliary cholangitis and primary sclerosing cholangitis is an extremely rare condition that has been reported in only few published cases so far in the literature. We highlight here the rarity of this condition and indicate the importance of its recognition.

**Case presentation:**

We report two cases showing the manifestations of both primary biliary cholangitis and primary sclerosing cholangitis in two Tunisian female patients aged 74 and 42 years, respectively. The first case is a woman who was initially diagnosed with decompensated cirrhosis. Magnetic resonance cholangiopancreatography showed multiple strictures of the common bile duct, and histological findings led to the diagnosis of primary biliary cholangitis/primary sclerosing cholangitis. She was successfully treated with ursodeoxycholic acid. The second case is a middle-aged woman, suffering from primary biliary cholangitis and who was treated with ursodeoxycholic acid. At her 12 month follow-up appointment, she presented with a partial clinical and biochemical response. Tests showed normal thyroid function, liver autoimmune tests for autoimmune hepatitis were negative, and celiac disease markers were also negative. The diagnosis of overlap syndrome of primary biliary cholangitis/primary sclerosing cholangitis was finally made on the results of magnetic resonance cholangiopancreatography that showed multiple strictures of the common as well as intrahepatic bile ducts. The patient was put on ursodeoxycholic acid at a higher dose.

**Conclusions:**

Our cases raise awareness for this rare condition and indicate the importance of recognizing a possible overlap syndrome, especially in patients with primary biliary cholangitis, to optimize treatment. We suggest considering the overlap syndrome of primary biliary cholangitis/primary sclerosing cholangitis when a patient presents with the diagnostic criteria of both diseases.

## Introduction

Overlap syndrome is a condition characterized by the coexistence of features belonging to different disorders within the spectrum of autoimmune liver diseases: autoimmune hepatitis (AIH), primary biliary cholangitis (PBC), and primary sclerosing cholangitis (PSC) [1]. The overlap syndromes of AIH with PBC and of AIH with PSC are well known. However, the overlap of PBC with PSC is rare and has been reported in only a few cases in the literature [2]. We describe here two cases of overlap syndrome between these two autoimmune cholestatic liver diseases to illustrate this rare condition, and through this illustration we will detail the clinical presentation and the therapeutic management.

## Case reports

### First case

**Fig. 1:**
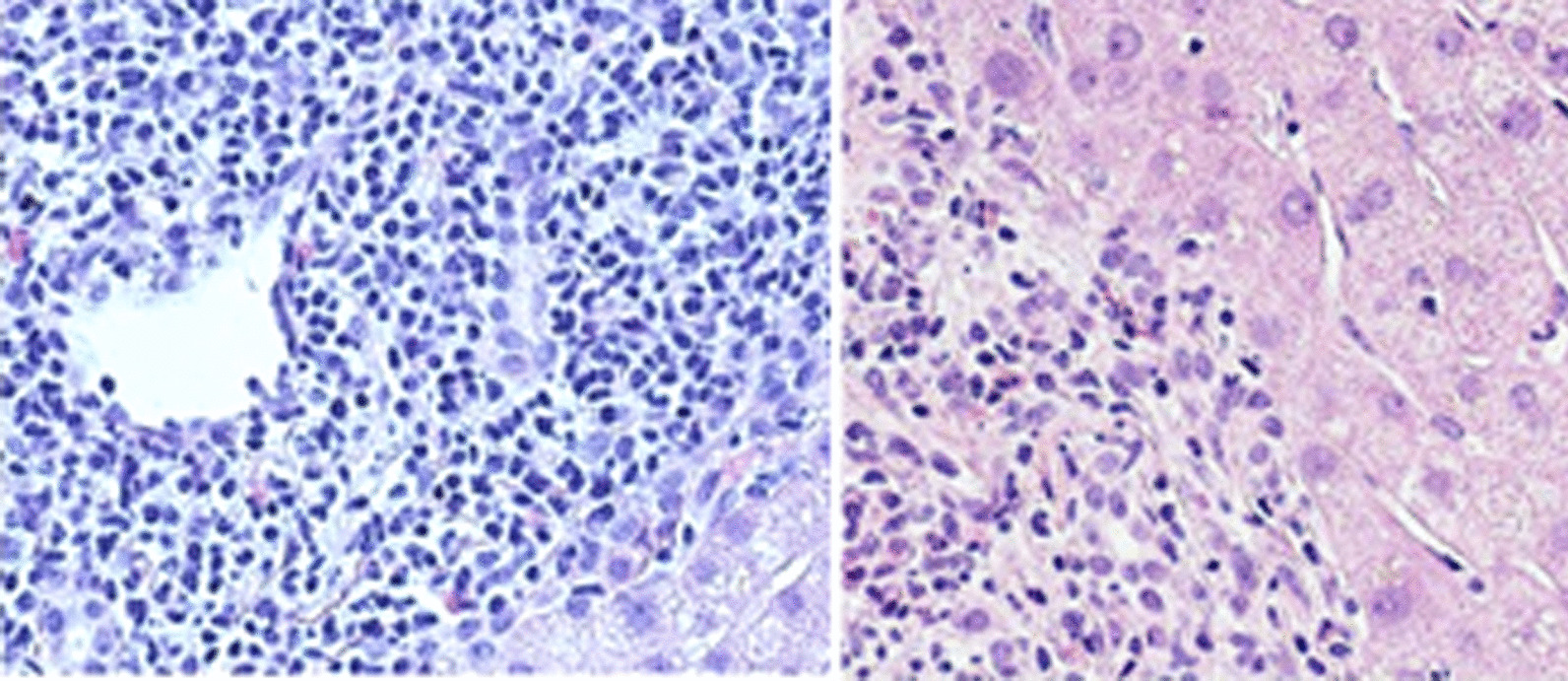
Lymphoplasmacytic inflammatory infiltrate with eosinophilic component with minimal piecemeal necrosis. Bile ducts were attacked in several tracts by lymphocytic cells, and periductal concentric fibrosis

**Fig. 2:**
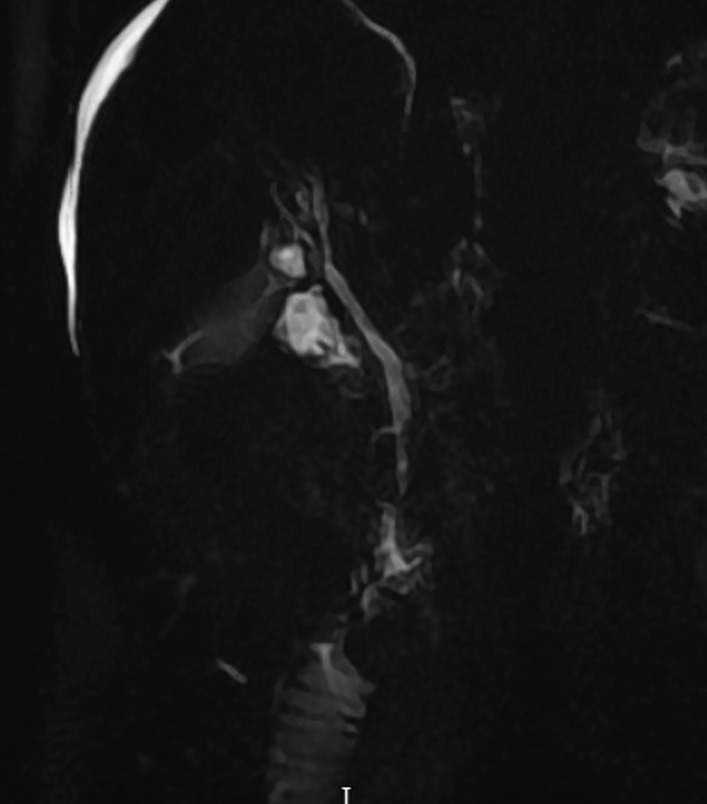
Cholangiopancreatography (magnetic resonance cholangiopancreatography): multiple strictures of the common bile duct with regular profile of the intrahepatic bile ducts

A 74-year-old Tunisian woman, with no significant medical history, was admitted to our department because of fatigue, abdominal distension, and appearance of jaundice in the last 2 weeks. There was no history of alcohol consumption, hepatotoxic medications, previous jaundice, or pruritis. On examination, she had pedal edema, icterus, ascites, and spider-like blood vessels on the skin. She had no hepatosplenomegaly, nor collateral circulation. Biological tests showed platelet count of 110 × 10^9^/L (reference range 150–400 × 10^9^/L), and prothrombin time was 70%. Her biochemical tests showed alkaline phosphatase (ALP) more than three times the upper limit of normal (ULN) (506 IU/L, ULN < 150 IU/L), gamma-glutamyl transferase (GGT) about seven times the ULN (273 IU/L, ULN < 36 IU/L), elevated levels of bilirubin total bilirubin/direct bilirubin (TB/DB: 24.6/20 µmol/L, ULN < 20 µmol/L), and hypoalbuminemia (28 g/dL). Alanine aminotransferase (ALT) and aspartate aminotransferase (AST) were normal. Ultrasound examination showed morphological features of advanced liver disease without alterations of the biliary tract. Liver stiffness measured by Fibroscan was 25 kPa. There were grade II esophageal varices and GOV I gastric varices at the upper endoscopy. At this stage, the diagnosis of cirrhosis was considered. Serum tests for hepatitis B and C viruses were negative. Autoimmune profile tested by indirect immunofluorescence showed a positivity for anti-nuclear (ANA) (titer: 1:100, speckled) and anti-smooth muscle antibody without specificity to F-actin. The other autoimmune tests resulted negative, in particular anti-mitochondrial antibody (AMA). Immunoglobulin levels (IgM) were within the normal range (2.27, normal range 0.4–2.63), whereas her IgG level was slightly high (25.8, normal range 6.58–18.37). A liver biopsy was performed to complete the diagnostic workup. The liver tissue specimen showed a moderate lymphoplasmacytic inflammatory infiltrate with eosinophilic component with minimal piecemeal necrosis and evidence of significant fibrosis. Bile ducts were attacked in several tracts by lymphocytic cells; periductal concentric fibrosis of some of small interlobular bile ducts was found corresponding to a sclerosing cholangitis (Fig. 1). The overall picture showed chronic hepatitis features and biliary aggression without evidence of portal and lobular granulomas, hepatocyte rosetting, and steatosis. We performed magnetic resonance cholangiopancreatography (MRCP), which showed multiple strictures of the common bile duct with regular profile of the intrahepatic bile ducts (Fig. 2). On the basis of the clinical, immunological, histological, and radiological findings, the diagnosis of an overlap syndrome between PBC and PSC was retained. Colonoscopy was negative for inflammatory bowel disease. The patient was subsequently started on diuretics and ursodeoxycholic acid (UDCA) 18 mg/kg daily. On follow-up, her ascites had resolved after 4 weeks. ALP and GGT reduced to 161 U/L and 139 U/L, respectively, and she was continued on UDCA. No episodes of decompensation occurred over 12 months of follow-up.

### Second case

We present the case of a 42-year-old Tunisian female, with no significant medical history, who was referred to our ward in 2015 due to generalized pruritus, fatigue, and deranged liver function tests. There was no history of drug or alcohol intake. On examination, a hepatosplenomegaly as well as venous collateral circulation were found. At that time, liver function tests showed serum ALP levels about two times the upper limit of normal (305 IU/L, ULN < 150 IU/L), GGT about six times the upper limit of normal (221 IU/L, ULN < 36 IU/L), and AST and ALT levels about two times the upper limit of normal. Bilirubin levels were elevated (TB/DB: 32/25 µmol/L). Serum test for hepatitis C virus was negative. Hepatitis B markers were negative for HBs antigen; HBc and HBs antibodies were positive. The HBs antibodies level was about 235 IU/L. Hepatitis B viral was negative. Autoimmune profile showed a positivity for anti-nuclear (ANA) (titer: 1:600), antimitochondrial antibody (AMA), and anti-gp210 (+++). Levels of immunoglobulin were elevated for IgM 2.8 g/L (normal range, 0.4–2.3 g/L). On the basis of the clinical, biological, and immunological findings, the patient was diagnosed with PBC. She was treated with ursodeoxycholic acid (15 mg/kg/day). At a 12 month follow-up appointment, liver tests were still deranged (GGT and ALP about 5 times and 1.5 times the upper limit of normal, respectively). At this stage, tests showed normal thyroid function. Liver autoimmune tests for autoimmune hepatitis and celiac disease markers were also negative. Liver biopsy showed dense lymphoplasmacytic infiltrate and lymphoid aggregate in the portal tract, bile ducts were attacked by lymphocytic cells. There was no evidence of histologic features of PSC. Staging fibrosis according to the METAVIR score indicated portal fibrosis without cirrhosis (F3) and severe activity (A3).

**Fig. 3:**
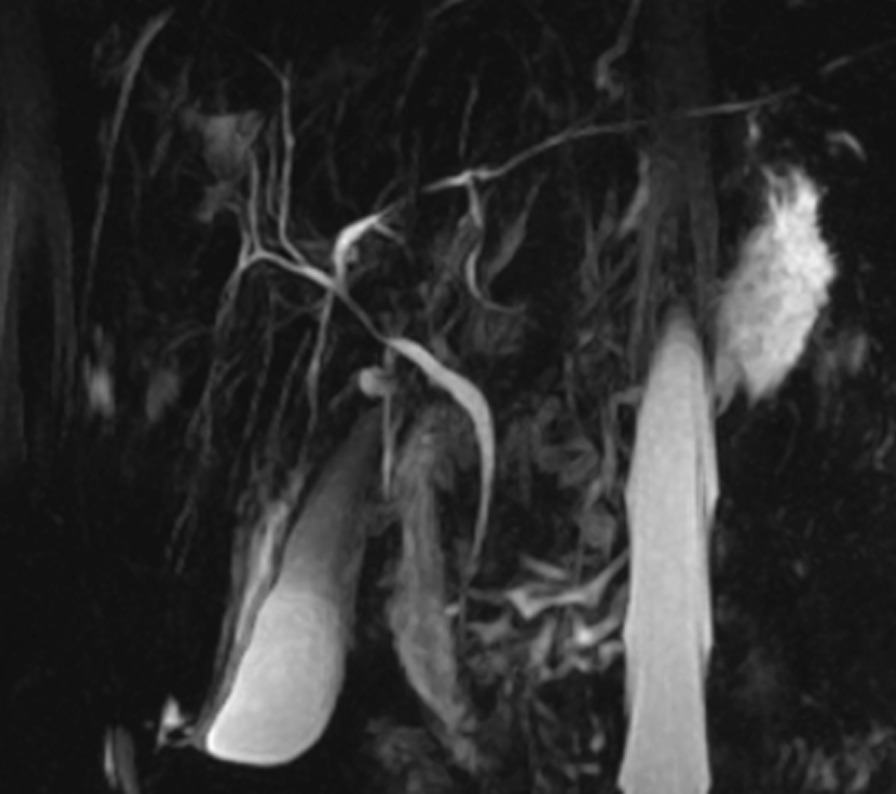
Magnetic resonance cholangiopancreatography: multiple strictures of the common bile duct and intrahepatic bile ducts

We performed MRCP, which showed multiple strictures of the common bile duct and intrahepatic bile ducts (Fig. 3). On the basis of the radiological findings, the diagnosis of PBC/PBC was made. The patient was treated with a high dose of UDCA (20 mg/kg/day). She improved clinically and biologically; ALP and GGT dropped to 171 and 113, respectively, within 2 months.

## Discussion

Overlap syndrome means the association of two or more autoimmune liver diseases in the same person, and can occur simultaneously or sequentially [2]. AIH/PBC overlap syndrome is the most studied and the most frequent of these disorders. Its prevalence is approximately 2% in adults initially diagnosed as AIH [3], and it occurs in 10–20% of patients with PBC [4]. The prevalence of AIH/PSC has been estimated to 6–14% of adults with PSC [4] and depends upon the presence (44%) or absence (8%) of associated inflammatory bowel disease among adults with AIH [5]. PBC/PSC has also been reported in few cases in the literature. However, due to the small sample size of these overlap cases, the prevalence is still undetermined [6]. A literature review identified ten cases until 2020 [7]. The importance of describing the two cases of overlap syndrome between these two autoimmune cholestatic liver diseases is to detail the clinical presentation and the therapeutic management.

The pathogenesis of overlap syndromes has been explained by different hypotheses. They could represent classic diseases with atypical manifestations, transition stages in the evolution of classic diseases, or two diseases in the same individual. Finally, it has been suggested that overlap syndromes are separate pathological entities with their own distinctive pathogenic mechanisms, and clinical, biochemical, histological, and evolutionary outcomes [8]. Ninety percent of  PBC cases are women, whereas  PSC is more common in men. Overlap syndrome PBC/PSC is seen more often in women; indeed, nine of the ten cases reported are females.

Both PSC and PBC are responsible of typical common symptoms such as jaundice, pruritis, and fatigue, which are due to cholestase [9]. However, most patients are asymptomatic in the early course of the disease [6]. Indeed, in the different studies of overlap syndromes of PBC/PSC described in the literature, up to 50% of patients were asymptomatic at the time of diagnosis and PBC was diagnosed on the basis of the biochemical and immunological findings [2, 10–12].

The diagnosis of PBC can be established if two of three objective criteria are present: unexplained elevated ALP ≥ 1.5 times the upper normal value for at least 6 months, serum AMA titers ≥ 1:40, or if AMA negative, positivity of anti-sp100/anti-gp 210 and compatible liver histology. Although liver biopsy is no longer required for the diagnosis of PBC, in many cases it is very helpful, especially when an overlap syndrome is evoked. The typical lesion is the non-suppurative cholangitis and interlobular bile duct injury [4, 7]. Elevated immunoglobulin M (IgM) levels are also seen.

There is no specific biological or immunological marker for the diagnosis of PSC. The fibro-obliterative lesion is the histological hallmark. It begins as concentric rings of fibrosis, known as onion skinning, around bile ducts. However, this is not always seen, but it supports the diagnosis of PSC when present. With time, this concentric fibrosis squeezes off and obliterates the bile duct lumen, leaving behind a fibrous plug or scar. There is often a mild lymphocytic infiltrate that accompanies the fibrosis, although the inflammation is much less prominent than in PBC [13]. Although unlikely, diagnosis of PSC can also be made by typical cholangiographic findings and the exclusion of secondary causes. The typical MRCP are diffuse, multifocal, short segmental strictures and mild dilatation in the intrahepatic as well as extrahepatic bile ducts alternating with normal ducts. As the strictures worsen, the peripheral  bile ducts become poorly visualized on MRCP ("pruned tree" appearance) [14].

There are no defined criteria available for PBC/PSC overlap syndrome, which suggests that this syndrome is diagnosed when a patient presents with the association of the diagnostic criteria of both diseases. PBC/PSC is generally characterized by a cholestatic laboratory profile, AMA positivity or other antibody specific to PBC and/or histologic characteristics of PBC, and cholangiographic and/or histologic features of PSC [6]. In the cases we reported, the diagnosis of overlap syndrome of PBC/PSC was made in a patient who was already being followed for PBC and who subsequently presented the radiological criteria for PSC in one case. In the other case, diagnosis was made on the basis of histological findings in a cirrhotic patient. To retain the diagnosis of associated PSC to PBC already diagnosed, we will take into consideration histological and/or radiological criteria.

The first-line therapy for PBC and PSC is based on UDCA given at a dose of 13–15 mg/kg/day in the case of PBC and 18–20 mg/kg/day in the case of PSC. The benefit of UDCA is proved in PBC. However, in PSC,  UDCA is responsible only for a biological improvement, without any effect on histological lesions [15]. There is currently no effective therapy for PSC that affects mortality, other than liver transplantation indicated for end-stage cirrhosis [16]. Furthermore, no standardized management has been formulated. Treatment of overlap syndrome of PBC/PSC is based on a higher dose of UDCA (18–20 mg/kg/day). Endoscopic dilation and stenting is important in good biliary drainage. Liver transplantation is indicated at patients with end-stage complications including decompensated cirrhosis, refractory ascites, recurrent encephalopathy, and severe pruritis.

It is important to be aware that the development of PSC overlap may be responsible for the worsening in cholestasis or primary/secondary non response to UDCA treatment in PBC patients previously responsive to UDCA.

## Conclusion

A review of the literature and the two reported cases contribute to better defining this rare variant autoimmune liver disease syndrome, and raise awareness of the importance of correctly recognizing this syndrome for treatment optimization. We suggest retaining the diagnosis when a patient presents with the association of the diagnostic criteria of both diseases.

## Data Availability

The data that support the findings of this study are available on request from the corresponding author (HY). The data are not publicly available due to restrictions (for example, they contain information that could compromise the privacy of research participants).
